# On the Theory and Practice of Midwifery

**Published:** 1860-10

**Authors:** 


On the Theory and Practice of Midwifery. By Fleetwood Churchill, M. D., M. R. I. A., &c., &c., &c. With additions by D. Francis Condie, M. D., &c. A new American from the fourth corrected and enlarged English edition. Philadelphia: Blanchard & Lea, 1860.

The old students of "Churchill's System" will scarce recognize in the present portly volume of six hundred and fifty-odd pages, closely but clearly printed, something smaller, but as distinctly-typed matter, the familiar volume of 18 and 18 ; and which he prizes with his "Wilson," "Wood" and "Carpenter"—the very "household words" of his medical vocabulary.

But with its growth of bulk its value has increased; and if he set much store by it in his early day, he will prize it none the less in its maturity and fullness, now that he, himself, has waxed somewhat more pondrous, mentally and physically.

We are not sure but it may be supererogatory to enter at this time into the details of what must be an almost unqualified approbatory criticism; for the merits of Dr. Churchill's work as a text-book and epitome of obstetrics, its exceeding richness in statistics, its fullness of detail and general accuracy, and its author's prominence in the branch he has made the speciality of a long life, are well known to the profession.

The careful and elaborate revision, however, the important addition, and thorough posting-up to the present time, of everything especial or noteworthy in obstetric science, as well by the American editor as by the English author, warrant us in, at least, calling attention to it.

The most notable additions to the present volume, are the chapters entitled "*Obstetric Moraltiy*" and "*Qualifications and Duties of Monthly Nurses*"—the latter being a collation of extracts from Churchill's "Manual for Midwives and Monthly Nurses," by the American editor, and not found in the Dublin imprint. The former is an essay on the operation of craniotomy, the result of a controversy upon the subject, which occurred during 1858, between Churchill and a writer in "*The Dublin Review.*" The treatise is exhaustive, logical and satisfactory, settling definitely the mooted point which has distressed many very worthy people, who have allowed religious prejudice to overcome their common sense.

Dr. Churchill claims, and we think, truly, that no English author has hitherto entered so fully into the subject.

